# Nonlinear association of serum uric acid and C-peptide with arterial stiffness in patients with type 2 diabetes: a real-world study

**DOI:** 10.3389/fendo.2026.1700359

**Published:** 2026-02-12

**Authors:** Youming He, Yufeng Jin, Mengqi Gao, Jing Jin, Ke Chen, Lixin Zhou, Jingbin Shi, Bei Luo, Yongqian Liang

**Affiliations:** 1 Department of Epidemiology and Health Statistics, School of Public Health, Xinjiang Second Medical College, Karamay, China; 2 Guangdong Institute of Gastroenterology, The Sixth Affiliated Hospital, Sun Yat-sen University, Key Laboratory of Human Microbiome and Chronic Diseases (Sun Yat-sen University), Ministry of Education, Guangzhou, China; 3 Department of Preventive Medicine, School of Public Health, Xinjiang Second Medical College, Karamay, China; 4 Department of Nasopharyngeal Carcinoma, Sun Yat-sen University Cancer Center, Guangzhou, China; 5 The Eighth Affiliated Hospital of Southern Medical University, The First People's Hospital of Shunde, Foshan, China; 6 Department of Endocrinology and Metabolism, The Eighth Affiliated Hospital of Southern Medical University, Shunde Hospital, Southern Medical University (The First People's Hospital of Shunde, Foshan), Foshan, China

**Keywords:** arterial stiffness, C-peptide, cross-sectional study, diabetes, uric acid

## Abstract

**Background:**

Diabetes has become one of the most serious and prevalent chronic diseases, and its cardiovascular complications are responsible for over 50% of diabetes-related deaths. However, the relationships between uric acid (UA) and C-peptide on arterial stiffness (AS) in patients with type 2 diabetes mellitus (T2DM) are still poorly understood. This study aimed to evaluate the associations between UA and C-peptide with AS in T2DM patients.

**Methods:**

In this cross-sectional study of 1,715 participants with T2DM, we recorded levels of fasting UA, C-peptide, and other characteristics. Elevated AS was defined as a brachial-ankle pulse wave velocity (baPWV) of ≥1,400 cm/s. Logistic regression and a restricted cubic spline (RCS) model were employed to assess the associations of UA and C-peptide with AS.

**Results:**

Fasting UA and C-peptide levels were independently and significantly associated with elevated AS in patients with T2DM, as determined by multivariate analyses (*P* < 0.05). Notably, RCS analyses revealed nonlinear relationships with threshold effects between fasting UA, C-peptide, and elevated AS (P for nonlinearity < 0.05). Compared to patients with C-peptide levels < 0.580 μg/L, those with levels ≥ 0.580 μg/L had an approximately 87% relatively higher odds of elevated AS (OR = 1.87, 95% CI: 1.32, 2.65).

**Conclusion:**

The elevated AS odds in patients with T2DM were nonlinearly associated with the levels of serum fasting UA and C-peptide.

## Introduction

Diabetes has emerged as one of the most serious and prevalent chronic diseases in recent years (1). Global epidemiological data from the 10th edition of the International Diabetes Federation (IDF) Diabetes Atlas revealed a prevalence of 537 million adults with diabetes in 2021, with China shouldering the largest burden (140 million cases), a figure anticipated to increase by 24% to 174 million within 25 years (2). Notably, diabetes mellitus significantly increases cardiovascular disease (CVD) risk (3, 4), and cardiovascular complications constitute the leading cause of death, responsible for more than 50% of diabetes-associated mortality (5).

Current evidence demonstrates that diabetes significantly elevates CVD risk, primarily through the progression of arterial stiffness (AS)—an independent predictor of cardiovascular events (6, 7). The pathophysiology of AS involves vascular endothelial dysfunction, arterial wall thickening, and reduced vascular elasticity (8). These structural changes decrease arterial compliance and impair diastolic function (9). Extensive research has established significant associations between conventional CVD risk factors and AS development in diabetes (3, 4). Notably, early intervention targeting low-density lipoprotein cholesterol (LDL-C), triglycerides (TG), and waist circumference (WC) can mitigate AS progression in type 2 diabetes mellitus (T2DM) (10), highlighting the particular vulnerability of diabetic patients with coexisting cardiovascular risk factors.

Emerging evidence implicates serum uric acid (UA) as a novel metabolic risk factor for CVD (11, 12). As the end product of purine metabolism in humans, elevated UA levels (hyperuricemia) result from disorders in purine nucleotide metabolism (13–15). Hyperuricemia demonstrates strong associations with elevated plasma triglyceride levels and atherogenic indices (16, 17). While multiple studies have established UA’s role in vascular dysfunction and atherosclerotic progression (18), its relationship with AS remains understudied.

As a 31-amino acid peptide derived from pancreatic proinsulin cleavage, C-peptide is secreted in equimolar amounts with insulin (19). Its circulating concentration serves as a reliable biomarker for assessing endogenous insulin secretion, β-cell function, and insulin resistance (20–23). Elevated levels of C-peptide are observed in individuals with insulin resistance and early T2DM (24). The influence of C-peptide concentration on diabetes patients is still controversial. While some studies associate low C-peptide levels with increased CVD risk in T2DM patients (25), others report that elevated baseline C-peptide predicts higher all-cause and cardiovascular mortality in newly diagnosed T2DM (26).

Existing research has indicated correlations between hyperuricemia and insulin resistance (27–29). Elevated UA levels correlate with increased C-peptide concentrations, and prediabetic individuals with high C-peptide levels exhibit greater susceptibility to gout development (30). The relationship between elevated AS risk and varying strata of serum C-peptide levels in T2DM patients has not been clearly established. Based on this, we postulated that UA and C-peptide exhibit nonlinear associations with the odds of elevated AS. Accordingly, a real-world observational study was carried out to investigate the associations between different levels of UA and C-peptide and elevated AS in a T2DM population.

## Methods

### Study design and ethics

**Figure 1:**
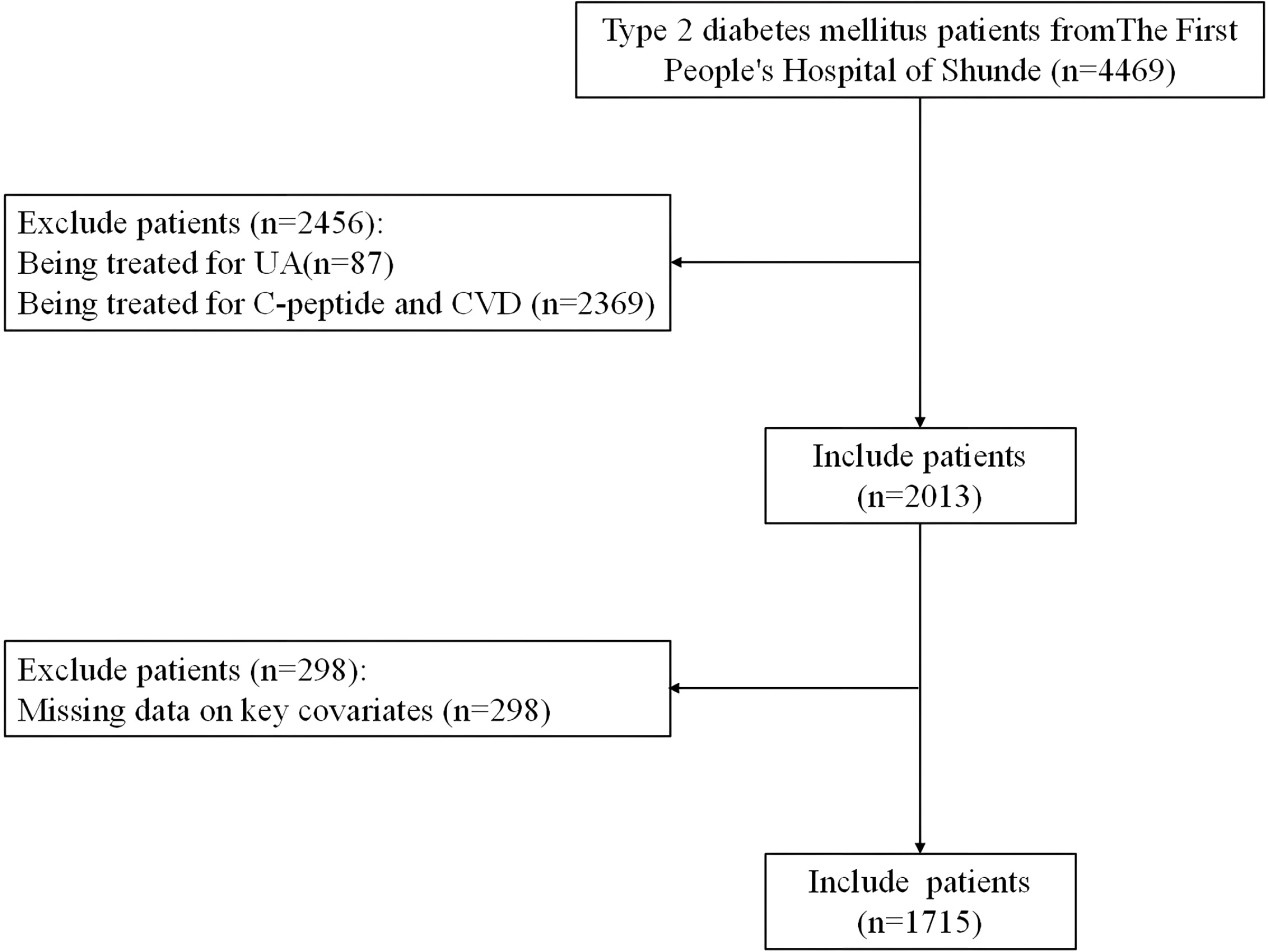
Flowchart of participants selection.

We conducted a real-world cross-sectional study at The First People’s Hospital of Shunde (Foshan, China) from June 2020 to September 2022, enrolling 4,469 patients with T2DM. All participants underwent standardized physical examinations, clinical assessments, and questionnaire surveys. Exclusion criteria comprised patients receiving UA-lowering therapy (*n* = 87), those on medications affecting C-peptide levels or CVD (*n* = 2,369), and cases with missing critical data (UA, C-peptide, or brachial-ankle pulse wave velocity [baPWV]) (*n* = 298). The final analysis included 1,715 eligible participants. A detailed study flowchart is provided in **Figure 1**.

The study protocol was approved by the Medical Ethics Committee of The First People’s Hospital of Shunde and adhered to the principles of the Declaration of Helsinki. As a retrospective analysis using anonymized hospital system data (without personal identifiers), written informed consent was waived.

### Basic information collection, anthropometric assessment, and laboratory measurements

All participants completed structured questionnaires to collect demographic and lifestyle data, including age, sex, education level, smoking status, alcohol consumption, medication use, and physical activity patterns. Smoking status was categorized as current smokers (regular smoking at the time of survey) or non-smokers (including never-smokers and former smokers who had quit for at least 6 months). Alcohol consumption was similarly classified as current drinkers (consumption at least once per week) or never drinkers (no regular alcohol intake). Trained nurses conducted standardized anthropometric measurements using calibrated instruments, with height measured to the nearest 0.1 cm and weight to the nearest 0.1 kg while participants wore light clothing without shoes. Body mass index (BMI) was calculated as weight in kilograms divided by height in meters squared. Trained nurses measured waist circumference (at the midpoint between the lower rib margin and the iliac crest) and hip circumference (at the level of the greater trochanter) to the nearest 0.1 cm using calibrated measuring tapes at the end of normal expiration. Waist-to-hip ratio (WHR) was calculated as waist circumference divided by hip circumference. After at least 15 min of seated rest, systolic (SBP) and diastolic (DBP) blood pressure were measured in triplicate with 1-min intervals using validated automated devices.

All blood samples were collected under standardized conditions: venipuncture was performed after a 12h overnight fast, and participants were instructed to take their regular medications as prescribed. Samples were transported under refrigerated conditions to a College of American Pathologists (CAP)–certified central laboratory within 2h–4h for analysis, and all biochemical assessments were conducted in the same laboratory using standardized methods.

### Study variables and outcome measures

The exposure variables were serum UA and C-peptide levels. The primary outcome was elevated AS, assessed through baPWV measurements using an Omron BP-203RPE III device (Kyoto, Japan). Following a 5-min supine rest, this validated method automatically recorded bilateral pressures and pulse waves, with baPWV calculated as the pulse wave travel distance divided by transit time. An elevated AS was defined as baPWV ≥1,400 cm/s, with final values representing the mean of left and right measurements. Elevated baPWV values indicate both increased AS and elevated CVD risk (31).

### Statistical analysis

A total of 1,715 subjects were stratified by AS status (normal/elevated) and categorized into quartiles based on UA and C-peptide levels, with continuous variables expressed as mean ± standard deviation (SD) for normal distributions or median (interquartile range, IQR) for non-normal distributions, and categorical variables presented as frequencies (percentages). Statistical comparisons employed one-way analysis of covariance (ANCOVA) for normally distributed continuous variables, the Kruskal–Wallis test for non-normally distributed continuous variables, and Pearson’s chi-square or Fisher’s exact test for categorical variables, with Bonferroni correction for multiple testing.

Adjusted logistic regression analyses were performed to evaluate the associations between UA/C-peptide levels and elevated AS odds. Three hierarchical models were constructed: Model 1 adjusted for age and sex; Model 2 included Model 1 covariates plus SBP, WHR, aspartate aminotransferase (AST), gamma-glutamyl transferase (GGT), and high-density lipoprotein cholesterol (HDL-C); and Model 3 further incorporated Model 2 covariates with additional adjustments for current alcohol consumption, smoking status, education level, and family history of diabetes. Quartiles were modeled as ordinal variables for trend testing, with odds ratios (ORs) and 95% confidence intervals (CIs) calculated per 1-unit increase in exposure variables. Nonlinear relationships were examined using restricted cubic splines (RCS) with knots at the 10th, 50th, and 90th percentiles. All statistical analyses were conducted using R software (version 4.2.2; https://www.R-project.org), with two-tailed *P*-values < 0.05 considered statistically significant.

## Result

### Characteristics of participants

The final analysis included 1,715 participants (1,156 [67.4%] with elevated AS), with a mean age of 51.07 ± 12.42 years. Median (interquartile range) serum C-peptide levels were 1.55 (1.00–2.44) μg/L in normal AS versus 1.81 (1.14–2.58) μg/L in elevated AS, while UA levels were 309.00 (241.55–400.50) μmol/L in normal AS versus 330.50 (258.75–400.00) μmol/L in elevated AS. Baseline characteristics stratified by AS status are summarized in **Table 1**. **Supplementary Tables S1**, **S2** display baseline characteristics of participants by UA and C-peptide quartiles, showing that both third quartiles of UA and C-peptide were associated with higher baPWV levels.

**Table 1:** Baseline characteristics according to AS status.

Characteristics	Normal AS (*n* = 559)	Elevated AS (*n* = 1156)	*P*-value
Age (years)	50.31 ± 12.16	51.44 ± 12.53	0.08
Male (%)	384 (68.69%)	771 (66.70%)	0.44
Education (%)			0.22
High school or below	240 (42.93%)	453 (39.19%)	
Above high school	319 (57.07%)	703 (60.81%)	
BMI (kg/m^2^)	25.36 ± 3.94	25.35 ± 3.91	0.95
WHR (%)	0.93 ± 0.07	0.94 ± 0.07	0.35
SBP (mmHg)	132.10 ± 18.95	133.12 ± 19.74	0.31
DBP (mmHg)	79.41 ± 11.61	79.23 ± 11.85	0.77
current smoking (%)	164 (29.34%)	314 (27.16%)	0.43
current drinking (%)	209 (40.90%)	387 (36.89%)	0.14
Family history of diabetes (%)	234 (41.86%)	465 (40.16%)	0.53
ALT (U/L)	24.00 (17.00,36.00)	25.00 (17.00,37.00)	0.80
AST (U/L)	19.00 (16.00,26.00)	21.00 (17.00,27.00)	< 0.01
GGT (U/L)	27.00 (18.00,41.25)	29.00 (19.00,48.00)	< 0.01
TG (mmol/L)	1.55 (1.02,2.50)	1.57 (1.12,2.42)	0.39
TC (mmol/L)	5.11 (4.36,6.00)	5.07 (4.28,6.01)	0.36
HDL-C (mmol/L)	1.15 (0.97,1.37)	1.17 (0.99,1.40)	0.13
LDL-C (mmol/L)	2.92 (2.42,3.49)	2.91 (2.36,3.50)	0.42
FPG (mmol/L)	8.42 (6.75,12.53)	8.66 (6.45,12.42)	0.61
insulin (μU/ml)	8.05 (4.80,13.02)	8.38 (5.09,13.20)	0.58
UA (μmol/L)	309.00 (241.55,400.50)	330.50 (258.75,400.00)	0.02
C-peptide (μg/L)	1.55 (1.00,2.44)	1.81 (1.14,2.58)	< 0.001

Data are presented as mean ± SD, median (IQR), or number (%), as appropriate.

AS, arterial stiffness; SD, standard deviation; IQR, interquartile range; SBP, systolic blood pressure; DBP. diastolic blood pressure; BMI, body mass index; WHR, waist–hip ratio; FPG, fasting plasma glucose; ALT, Alanine aminotransferase; AST, Aspartate aminotransferase; GGT, γ-glutamyl transpeptidase; TG, triglycerides; TC, total cholesterol; HDL-C, high-density lipoprotein cholesterol; LDL-C, low-density lipoprotein cholesterol; UA, uric acid.

### The association of UA and C-peptide with AS

After multivariable adjustment, both elevated UA and C-peptide levels showed significant associations with increased AS risk in T2DM patients (*P* for trend < 0.01 for both biomarkers, **Table 2**). Each unit increase in UA was marginally associated with higher AS risk (OR = 1.00, 95% CI: 1.00–1.00, *P* = 0.05), while each unit increase in C-peptide demonstrated a stronger association (OR = 1.04, 95% CI:1.02–1.07, *P* < 0.001). In fully adjusted models, the third and fourth UA quartiles showed significantly elevated AS risk versus Q1 (Q3: OR = 1.14, 95% CI: 1.07–1.23, P < 0.001; Q4: OR = 1.07, 95% CI: 1.01–1.16, *P* = 0.02), with similar patterns observed for C-peptide (Q3: OR = 1.13, 95% CI: 1.06–1.21, *P* < 0.001; Q4: OR = 1.08, 95% CI: 1.01–1.15, *P* = 0.03).

**Table 2:** The risk for elevated AS according to serum UA and C-peptide concentrations.

Study variables	Model 1		Model 2		Model 3	
	OR (95% CI)	*P*	OR (95% CI)	*P*	OR (95% CI)	*P*
UA (μmol/L)						
per unit increment	1.00 (1.00,1.00)	0.09	1.00 (1.00,1.00)	0.05	1.00 (1.00,1.00)	0.05
Quartile 1 (264/433)	1.00 (Reference)	–	1.00 (Reference)	–	1.00 (Reference)	–
Quartile 2 (284/426)	1.06 (0.99,1.13)	0.07	1.94 (1.00,1.13)	0.05	1.07 (1.00,1.14)	0.05
Quartile 3 (322/430)	1.15 (1.08,1.22)	< 0.001	1.15 (1.08,1.23)	< 0.001	1.14 (1.08,1.23)	< 0.001
Quartile 4 (286/426)	1.06 (1.00,1.13)	0.06	1.07 (1.00,1.14)	0.04	1.07 (1.01,1.16)	0.02
P for trend		< 0.01		< 0.01		< 0.01
C-peptide (μg/L)						
per unit increment	1.04 (1.02,1.06)	< 0.001	1.04 (1.02,1.06)	< 0.001	1.04 (1.02,1.07)	< 0.001
Quartile 1 (275/430)	1.00 (Reference)	–	1.00 (Reference)	–	1.00 (Reference)	–
Quartile 2 (269/431)	0.99 (0.93,1.06)	0.85	0.99 (0.93,1.05)	0.07	1.01 (0.94,1.07)	0.09
Quartile 3 (315/428)	1.10 (1.04,1.18)	< 0.01	1.11 (1.04,1.18)	< 0.01	1.13 (1.06,1.21)	< 0.001
Quartile 4 (297/426)	1.06 (1.00,1.13)	0.05	1.06 (0.99,1.12)	0.06	1.08 (1.01,1.15)	0.03
P for trend		< 0.01		< 0.01		< 0.01

AS, arterial stiffness; UA, uric acid; OR, odds ratios; CI, confidence intervals.

Model 1: was adjusted age and gender.

Model 2: was adjusted model 1 covariates plus SBP, WHR, AST, GGT and HDL-C.

Model 3: was adjusted model 2 covariates plus current drinking, current smoking, education and family history of diabetes.

### RCS analysis based on logistic regression

**Figure 2:**
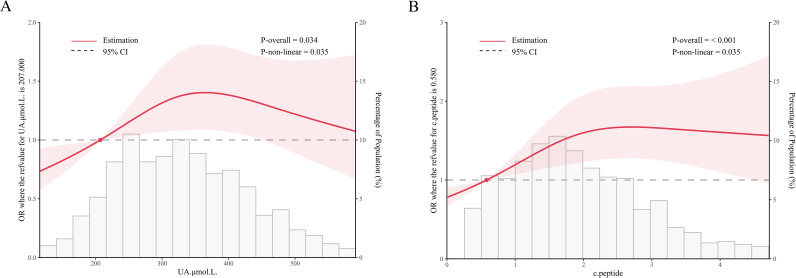
Nonlinearity association between UA, C-peptide level and elevated AS. RCS model was used to assess “the overall” and the “nonlinearity” association between UA, C-peptide level and elevated AS odds. Adjustments were made for age, gender, SBP, WHR, AST, GGT, HDL-C, current drinking, current smoking, education and family history of diabetes. **(A)** nonlinear relationship between UA and elevated AS odds; **(B)** nonlinear relationship between C-peptide and elevated AS odds.

RCS analyses revealed nonlinear relationships for both biomarkers (*P* for nonlinearity: UA, *P* = 0.034; C-peptide, *P* < 0.001), with UA showing risk escalation below 350 μmol/L (OR = 1 cutoff: 207 μmol/L) and C-peptide demonstrating analogous trends (OR = 1 cutoff: 0.580 μg/L, **Figure 2**).

### Threshold effects of UA and C-peptide for AS

The interval effects of fasting UA and C-peptide for elevated AS odds were evaluated using logistic regression analysis (**Table 3**). After adjusting for covariates, the increase of elevated AS odds in the subjects with UA ≥207 μmol/L had no statistical significance, compared with patients with UA <207 μmol/L. After adjusting for age, gender, SBP, WHR, AST, GGT, HDL-C, current drinking, current smoking, education, and family history of diabetes, the odds of elevated AS in the patients with C-peptide ≥0.580 μg/L relatively increased by about 87%, compared with that in the patients with C-peptide <0.580 μg/L (OR = 1.87, 95% CIs = 1.32, 2.65).

**Table 3:** Threshold effects of UA and C-peptide for AS.

Study variables	Model 1		Model 2		Model 3	
	OR (95% CI)	*P*	OR (95% CI)	*P*	OR (95% CI)	*P*
UA (μmol/L)						
< 207	1.00 (Reference)	–	1.00 (Reference)	–	1.00 (Reference)	–
≥ 207	1.12 (0.80, 1.56)	0.510	1.12 (0.79, 1.58)	0.518	1.07 (0.73, 1.53)	0.737
C-peptide (μg/L)						
< 0.580	1.00 (Reference)	–	1.00 (Reference)	–	1.00 (Reference)	–
≥ 0.580	1.92 (1.39, 2.64)	< 0.001	1.90 (1.37, 2.63)	< 0.001	1.87 (1.32, 2.65)	< 0.001

AS, arterial stiffness; UA, uric acid; OR, odds ratios; CI, confidence intervals.

Model 1: was adjusted age and gender.

Model 2: was adjusted model 1 covariates plus SBP, WHR, AST, GGT and HDL-C.

Model 3: was adjusted model 2 covariates plus current drinking, current smoking, education and family history of diabetes.

## Discussion

In the present study, we found that although UA and C-peptide were independently associated with the odds of elevated AS in patients with T2DM, the probability of elevated AS did not increase linearly with rising serum UA and C-peptide levels. Based on RCS analysis, the risk of elevated AS was nonlinearly associated with UA and C-peptide levels after adjusting for potential confounders. Specifically, when C-peptide levels were ≥ 0.580 μg/L, patients with T2DM appeared to have a relatively higher probability of elevated AS.

Although diabetes has more than 100 complications, more than half of diabetes deaths are caused by CVD (5). In this study, we observed a nonlinear association between UA and AS. While the RCS model suggested a potential inflection point around 207 µmol/L, this threshold did not achieve statistical significance in binary analysis. This may reflect that the vascular effect of UA follows a continuous and gradual pattern rather than an abrupt change at a precise cutoff, indicating that clinical evaluation of UA should focus on its continuous trend rather than relying on a single threshold. In alignment with our findings, the positive association between UA and AS was reported in previous cross-sectional and cohort studies (18, 32). In the relationship between UA and AS, UA inhibits the production of nitric oxide, leading to endothelial dysfunction (33). In addition, hyperuricemia stimulates the renin-angiotensin system, leading to excessive production of reactive oxygen species (ROS) (34). ROS causes vascular oxidative stress and endothelial dysfunction and inhibits the bioavailability of nitric oxide, which is associated with the risk of AS (35). On the other hand, UA also acts as an important free radical scavenger with antioxidant properties (36), which may explain the nonlinear association observed in this study, specifically, the plateau in the relationship between UA and AS beyond the inflection point. This antioxidant mechanism is consistent with reports of UA exhibiting protective effects in certain populations, such as cancer patients (37). Therefore, while UA may contribute to the pathophysiology of AS, its predictive value for macrovascular complications in patients with T2DM should be interpreted cautiously in light of this level-dependent, nonlinear relationship.

Several investigations have explored the link between fasting C-peptide concentrations and cardiovascular outcomes, revealing that elevated C-peptide levels are associated with an increased risk of cardiovascular complications in individuals with T2DM (38–41). The present study is the first to investigate the association between C-peptide and AS in patients with T2DM. This focus holds significant clinical relevance, as previous research on C-peptide and diabetic complications has primarily centered on microvascular pathologies (42), with limited attention to macrovascular complications—particularly atherosclerosis, which remains the leading cause of mortality in diabetic populations. Our findings provide evidence into the relationship between C-peptide and macrovascular complications in T2DM. The results demonstrated a positive association between fasting C-peptide levels and elevated AS risk, with further analysis revealing a nonlinear relationship. In nondiabetic individuals, several studies reported a positive linear association between C-peptide and AS markers (43, 44). Although previous studies have indicated that C-peptide levels in T2DM patients exhibit nonlinear relationships with glycemic control rates and renal dysfunction (45, 46), no prior research has specifically elucidated the association between C-peptide levels and AS in patients with T2DM.

The observed nonlinear relationship suggests a complex biological mechanism underlying the role of C-peptide in AS. At lower concentrations, increased C-peptide may reflect residual β-cell function and exert protective anti-inflammatory and vasoprotective effects via activation of the AMP-activated protein kinase α (AMPKα) pathway, which could potentially slow AS progression (47, 48). However, beyond a certain threshold, elevated C-peptide may promote AS by enhancing inflammatory responses (49), stimulating vascular smooth muscle cell proliferation (50, 51), or exacerbating insulin resistance. This dual role aligns with the previously described “double-edged sword” effect of C-peptide in diabetic complications (52, 53). Therefore, normal concentrations of C-peptide may exert a protective effect, whereas excessive C-peptide delivery may not confer additional benefits and could even be harmful. In the present study, the mean fasting C-peptide level was 1.55 ng/ml, and the turning point was observed at 0.58 ng/ml among participants with T2DM. In comparison, previous studies have reported different turning points regarding the association between C-peptide and cardiovascular risk in nondiabetic adults and patients with newly diagnosed T2DM (40). This indicates that both the normal physiological range of fasting C-peptide and its turning point require further estimation in future research.

The highlights of the present study include the conduct of a cross-sectional analysis that clarified the nonlinear associations between fasting C-peptide, UA, and the prevalence of elevated AS in patients with T2DM. However, several limitations should be considered. First, the study enrolled participants from a single hospital, which may limit the generalizability of the findings. Second, due to the cross-sectional design, it was not possible to establish a temporal sequence between UA or C-peptide levels and elevated AS; thus, the evidence provided is not conclusive. Therefore, larger-scale cohort studies are warranted. Third, although antidiabetic medications were accounted for, the impact of specific medication types on UA and C-peptide levels and subsequent AS risk was not examined. Importantly, our analysis did not include several clinically relevant variables, such as the duration of diabetes and more detailed measures of long-term glycemic control. These unmeasured or residual factors may confound the observed associations and should be considered when interpreting the results. Overall, this exploratory study underscores the need for and lays the groundwork for a prospective confirmatory study using an appropriately sized random sample to further validate the nonlinear relationship between new cases of elevated AS and UA or C-peptide levels.

## Conclusions

In summary, our findings indicate a nonlinear association of serum UA and C-peptide with elevated AS odds in patients with T2DM. When C-peptide ≥ 0.580 μg/L, patients with T2DM might have the relatively higher elevated AS odds. These findings highlight the potential relevance of fasting C-peptide and UA in assessing AS risk in this population. Further longitudinal studies are warranted to clarify their clinical utility and any possible implications for cardiovascular risk stratification.

## Data Availability

The raw data supporting the conclusions of this article will be made available by the authors, without undue reservation.
