# Supplementary material for: Nonlinear association of serum uric acid and C-peptide with arterial stiffness in patients with type 2 diabetes: a real-world study
Source: Front Endocrinol (Lausanne). 2026 Feb 12;17:1700359. doi: 10.3389/fendo.2026.1700359 (PMC12935652; doi:10.3389/fendo.2026.1700359)
Supplement: Supplementary file 1 [file Table1.docx]

| **TABLE S1 Baseline Characteristics according to quantiles of uric acid.** | | | | | |
| --- | --- | --- | --- | --- | --- |
| **Characteristics** | **Quantile 1 n=433** | **Quantile 2 n=426** | **Quantile 3 n=430** | **Quantile 4 n=426** | **P-value** |
| Age (year) | 50.68 ±12.85 | 51.15 ±12.78 | 50.52 ±11.76 | 51.96 ±12.25 | 0.23 |
| Male n(%) | 276 (63.74) | 301 (70.66) | 291 (67.67) | 287 (67.37) | 0.43 |
| Education (%) |  |  |  |  | 0.68 |
| ≤ High school | 170 (39.26) | 181 (42.48) | 180 (41.86) | 162 (38.03) |  |
| > High school | 263 (60.74) | 245 (57.51) | 250 (58.14) | 264 (61.97) |  |
| BMI (kg/m^2^) | 25.28 ±4.09 | 25.09 ±3.64 | 25.56 ±3.88 | 25.48 ±4.03 | 0.21 |
| WHR (%) | 0.93 ±0.07 | 0.94 ±0.07 | 0.94 ±0.07 | 0.94 ±0.07 | 0.26 |
| SBP (mmHg) | 132.51 ±21.23 | 132.70 ±19.01 | 133.04 ±19.58 | 132.90 ±18.01 | 0.72 |
| DBP (mmHg) | 78.69 ±12.34 | 79.52 ±11.57 | 79.68 ±11.91 | 79.27 ±11.24 | 0.45 |
| current smoking n(%) | 122 (28.18) | 120 (28.17) | 115 (26.74) | 121 (28.40) | 0.96 |
| current drinking n(%) | 134 (34.01) | 151 (39.02) | 160 (40.61) | 151 (39.22) | 0.12 |
| ALT (U/L) | 21.00 (15.00,33.00) | 23.00 (17.00,35.00) | 26.00 (19.00,41.00) | 27.00 (18.00,41.00) | <0.001 |
| AST (U/L) | 19.00 (16.00,25.00) | 20.00 (16.50,25.00) | 21.00 (17.00,28.00) | 21.00 (17.00,28.00) | <0.001 |
| GGT (U/L) | 22.00 (16.00,36.00) | 27.00 (19.00,41.00) | 32.00 (22.00,52.00) | 34.00 (22.00,56.00) | <0.001 |
| TG (mmol/L) | 1.20 (0.90,1.73) | 1.52 (1.05,2.28) | 1.70 (1.20,2.63) | 1.95 (1.31,3.32) | <0.001 |
| TC (mmol/L) | 4.99 (4.22,5.82) | 5.04 (4.31,5.95) | 5.08 (4.35,5.99) | 5.28 (4.42,6.16) | <0.01 |
| HDL-C (mmol/L) | 1.29 (1.07,1.48) | 1.21 (1.00,1.44) | 1.13 (0.98,1.33) | 1.06 (0.91,1.26) | <0.001 |
| LDL-C (mmol/L) | 2.77 (2.20,3.34) | 2.93 (2.41,3.51) | 2.91 (2.45,3.48) | 3.06 (2.50,3.69) | <0.001 |
| baPWV (cm/s) |  |  |  |  | <0.01 |
| ≤ 1400 | 169 (39.03) | 142 (33.33) | 108 (25.12) | 140 (32.86) |  |
| > 1400 | 264 (60.97) | 284 (66.67) | 322 (74.88) | 286 (67.14) |  |
| Data are presented as mean ± SD, median (IQR), or number (%), as appropriate | | | |  |  |
| Abbreviations: SD standard deviation, IQR interquartile range, SBP systolic blood pressure, DBP diastolic blood pressure, BMI body mass index, WHR waist-hip ratio, ALT Alanine aminotransferase, AST Aspartate aminotransferase, GGT γ-glutamyl transpeptidase, TG triglycerides, TC total cholesterol, HDL-C high-density lipoprotein cholesterol, LDL-C low-density lipoprotein cholesterol, baPWV, Brachial-ankle pulse wave velocity. | | | | | |

| **TABLE S2 Baseline Characteristics according to quantiles of C-peptide.** | | | | | |
| --- | --- | --- | --- | --- | --- |
| **Characteristics** | **Quantile 1 n=438** | **Quantile 2 n=432** | **Quantile 3 n=419** | **Quantile 4 n=426** | **P-value** |
| Age (year) | 51.31 ±12.59 | 50.63 ±12.88 | 51.04 ±11.56 | 51.31 ±12.62 | 0.89 |
| Male n(%) | 277 (63.24) | 286 (66.20) | 291 (69.45) | 301 (70.66) | 0.01 |
| Education (%) |  |  |  |  | 0.04 |
| ≤ High school | 156 (35.62) | 181 (41.90) | 174 (41.53) | 182 (42.72) |  |
| > High school | 282 (64.38) | 251 (58.10) | 245 (58.47) | 244 (57.28) |  |
| BMI (kg/m^2^) | 25.18 ±3.89 | 25.42 ±4.08 | 25.50 ±3.98 | 25.33 ±3.71 | 0.55 |
| WHR (%) | 0.93 ±0.07 | 0.93 ±0.07 | 0.94 ±0.06 | 0.94 ±0.07 | 0.24 |
| SBP (mmHg) | 133.34 ±19.28 | 133.07 ±19.40 | 132.14 ±19.96 | 132.57 ±19.35 | 0.44 |
| DBP (mmHg) | 79.07 ±11.35 | 79.45 ±11.81 | 79.45 ±11.60 | 79.20 ±12.34 | 0.87 |
| current smoking n(%) | 127 (29.00) | 119 (27.55) | 115 (27.45) | 117 (27.46) | 0.89 |
| current drinking n(%) | 135 (34.01%) | 138 (35.57) | 154 (40.00) | 169 (43.33) | <0.01 |
| ALT (U/L). | 21.00 (15.00,29.00) | 21.00 (16.00,32.00) | 25.00 (18.00,37.00) | 32.00 (22.00,55.00) | <0.001 |
| AST (U/L) | 19.00 (16.00,25.00) | 19.00 (15.50,24.00) | 20.00 (17.00,26.00) | 23.50 (18.00,32.75) | <0.001 |
| GGT (U/L) | 21.00 (15.00,34.00) | 26.00 (18.00,43.00) | 30.00 (21.00,45.25) | 39.00 (26.00,62.00) | <0.001 |
| TG (mmol/L) | 1.20 (0.85,1.76) | 1.41 (0.98,2.12) | 1.71 (1.26,2.77) | 1.99 (1.35,3.29) | <0.001 |
| TC (mmol/L) | 4.98 (4.21,5.89) | 5.08 (4.34,5.98) | 5.09 (4.42,5.97) | 5.22 (4.29,6.13) | 0.04 |
| HDL-C (mmol/L) | 1.25 (1.01,1.47) | 1.22 (1.04,1.44) | 1.11 (0.97,1.31) | 1.10 (0.94,1.30) | <0.001 |
| LDL-C (mmol/L) | 2.87 (2.28,3.47) | 2.88 (2.34,3.46) | 2.92 (2.51,3.51) | 3.00 (2.40,3.59) | 0.03 |
| baPWV (cm/s) |  |  |  |  | <0.01 |
| ≤ 1400 | 159 (36.30) | 160 (37.04) | 111 (26.49) | 129 (30.28) |  |
| > 1400 | 279 (63.70) | 272 (62.96) | 308 (73.51) | 297 (69.72) |  |
| Data are presented as mean ± SD, median (IQR), or number (%), as appropriate | | | |  |  |
| Abbreviations: SD standard deviation, IQR interquartile range, SBP systolic blood pressure, DBP diastolic blood pressure, BMI body mass index, WHR waist-hip ratio, ALT Alanine aminotransferase, AST Aspartate aminotransferase, GGT γ-glutamyl transpeptidase, TG triglycerides, TC total cholesterol, HDL-C high-density lipoprotein cholesterol, LDL-C low-density lipoprotein cholesterol, baPWV, Brachial-ankle pulse wave velocity. | | | | | |
